# Experiments with Indian Hemp

**Published:** 1855-01

**Authors:** Kirtley Ryland

**Affiliations:** Keokuk, Iowa


### EXPERIMENTS WITH INDIAN HEMP, OR HASHISH.

BY KIRTLEY RYLAND, M. D., KEOKUK.

The Cannabis Sativa, from which Bang, Hashish, Churrus, and other intoxicating agents are made, is a native of India, but differs in no essential particular from the common hemp plant of the western States, which doubtless possesses the same qualities as an intoxicant.

In Hindostan, Arabia, Persia, and other countries of the East, Hemp has long been employed for the purpose of intoxication.— The parts used are the tops, and a resinous matter which exudes from the surface of the plant. The tops are known as Gunjah, and the resin as Churrus.

In this country, an extract of the dried tops is made, and in this form it is used in medicine. Its use by the physician is however very limited, and very little is known concerning its effect in disease. Dr. O'Shaughnessy, of Dublin, has recommended it for sundry diseases where narcotics are indicated. The United States Dispensatory says, "it resembles opium in its operation, but differs from that narcotic in not diminishing the appetite, checking the secretions or constipating the bowels.

Having thus given a brief account of the Hemp Plant, and the preparations of it, I will now relate my own experience concerning it.

In March, 1854, whilst residing in St. Louis, I had several conversations with a young medical friend concerning the remarkable properties of hemp, and we finally determined to try the effect of it in our own persons. Accordingly we fixed an evening, and he procured a quantity of the extract, of which we each took a portion about the size of a pepper corn, and repaired to the theatre, in order to have the accessories of music, lights, and beauty, to our enjoyment.

Some time having elapsed, and perceiving no effect, I took another portion the size of the first, while at the same time my friend swallowed 2 pills more of the same description. In a very little time, I felt a slight dryness of the throat, and then a singular but delightful sensation creeping over me, which increased rapidly until my entire body quivered with it. Casting up my eyes, the vast dome of the Varieties, with its pictured ceiling, seemed to swell and dilate above me, the lights multiplied themselves into a thousand brilliant and beauteous forms, and cast a soft rose tint over the entire theatre, now apparently enlarged to ten times its real size; whilst the swelling music, and the lovely features, and sparkling glances of the fairer portion of the audience, rendered trebly charming by the marvellous Hashish, completed a picture of enchantment perfectly intoxicating in its gorgeous beauty. Ah! many a fair lady would give a goodly heritage, to be as beautiful as she seemed to my intoxicated senses on that evening.

And now the curtain rose, upon what was to me a company of sylphs and graces, but which was really a squad of very ugly ballet girls, and presently the heroine of the ballet bounded upon the stage. It is impossible to concieve the wondrous effect that the graceful movements and animated face of this magnificent danseuse, (Mademoiselle P——d,) had upon me; I seemed to be transported to fairy land, and yielded myself up to the delusion, and the feeling of satisfied bliss it occasioned. After a short time, the influence of the drug passed off, and during the whole time I was under its influence, regular intermissions occurred, during which, the appearance of things became natural. At this time I began to experience intense thirst, but feeling an aversion to water I procured some oranges, and sucked them, and never shall I forget the sensation which the first mouthful of orange juice produced upon me. My sense of taste was exalted a thousand times above its natural acuteness, and as the juice flowed over my tongue and palate, it caused every fibre of them to thrill with the most intense pleasure. It was the acme of sensual bliss. All my senses soon acquired this preternatural acuteness, and impressions that before were scarcely noticed, now caused the most pleasurable emotions. Here I will speak of two of the most singular effects of Indian Hemp upon the mind: The first is the singular lengthening of time; events and actions that reallv occupied but a few moments, seemed to extend over hours, and yet there seemed no slowness of movement.

It is impossible for me to give a correct idea of this singular psychological phenomenon. It must be experienced, to be appreciated. The second effect of which I spoke, I can better illustrate with an example, than by description: I saw in an adjoining box a richly dressed and fashionable lady, whose hands I observed were of a very coarse and plebeian pattern. "Ah!" said I to myself, "there's a parvenu putting on aristocratic airs, but her hands betray her," and this simple and natural conclusion appeared to me a triumph of logical reasoning unapproachable in brilliancy and profundity! I was a long time in arriving at this conclusion, and as it gradually formed itself in my mind, I experienced the same feeling that must animate a philosopher when the light of some grand truth fraught with good to mankind, first dawns upon him. My mind was so occupied with sensations, as to be incapable of reasoning.

And now "a change came o'er the spirit of my dream." A laughing devil took possession of me; I laughed at everything; at what was grave and what was gay; at the ladies and at the men; at the pirouettes of the dancers, and then at nothing. One of the characters in the piece, a huntsman, whose part consisted in rushing in and interrupting the lovers at the most interesting moment, amused me greatly by his ludicrous pantomimic gestures, and his outlandish appearance. Every appearance of his caused in me a fresh burst of uncontrollable mirth, and I was on the point several times of rising and remonstrating with him upon his unseasonable interruptions and ridiculous conduct, but was restrained by my companion, who had, as yet, felt scarcely any effect from his dose of Hashish.

But little further change took place in my sensations, until we left the Theatre, when I became seized with a mortal terror. I thought I would never wake from the horrid trance I was in, save with dethroned reason, and I spoke to my friend of the hideous consequences of such an issue to our frolic, but the laughing humor was upon him, and my gloomy forebodings received no other response than loud laughter; consequently I left him, and proceeded home.

Scarcely had I laid down in bed, ere there seemed to settle on and around me a huge cloud of lurid fire, which parched my throat and almost consumed my body with its fervent heat, and still I shrank from water, and could only alleviate my intense thirst with juicy fruits, apples, oranges and limes; quantities of which I had convenient. I have before spoken of the exquisite refinement, and acuteness of the sense and taste, which hemp produces. In addition to this, the appetite it causes, is perfectly marvellous. From eight o'clock in the evening, until two in the morning, I was eating constantly, and when at length my exhausted commissariat *forced* me to cease, I still felt as hungry as when I began. I would also remark, *en passant*, that hemp is *decidedly* aphrodisiac.

Shortly after lying down, I fell into a state between sleep and waking, in which material objects were lost sight of, and their places taken by the creatures of my own distempered brain.

> "A thousand fantasies
> Began to throng into my memory,
> Of calling shapes, and beckoning shadows dire,
> And airy tougues that syllable men's names,
> And sands, and shores, and desert wildernesses."

Through the long watches of the night, a sad and endless throng of gastly forms trooped across the red wall of fire that encompassed me. There came the dead of the battle-field, their gaping wounds dripping with gore, and their faces distorted with the death-agony; and then the shipwrecked, with garments wet with brine and the seaweed clinging to their tangled hair; and then the victims of the pestilence, their ghastly faces blotched with plague-spots, and their glassy eyes turned with a stony and horrible stare upon me, as I lay thrilled with horror at the dreadful thought that I should soon make one of that procession of dead men, with none to tell how I had died. Weeks, nay months, seemed to have rolled by and still they passed before me, until at last a huge figure stopped in front of me. As he gazed upon me, his form dilated and his eyes flashed with rage, I shrank from him, but with one spring he was upon me, his skeleton fingers grasped my throat and dragged me to the edge of a precipice. I was helpless in his hands, and my parched throat could utter no cry as he hurled me into the black gulf below. Down, down I fell thousands of feet, until at last I became insensible and knew nothing more until I waked in the morning, dreadfully prostrated and unable to rise. The next day however found me as well as ever, with no desire to repeat the experiment.

What my companion's experiences were I do not very well know. He has often promised to write them out, but has never done so. To those who would know more of the wonders of hemp, I commend an article in Putnam's Magazine for April, 1854, entitled "Visions of Hashish." It is written by Bayard Taylor, Esq., and describes his experience in a manner worthy of the great De Quincey himself. But I should certainly advise no one to take it.

KEOKUK, IOWA, Oct. 27th, 1854. Iowa Med Jour

